# Neural versus pneumatic control of pressure support in patients with chronic obstructive pulmonary diseases at different levels of positive end expiratory pressure: a physiological study

**DOI:** 10.1186/s13054-015-0971-0

**Published:** 2015-06-09

**Authors:** Ling Liu, Feiping Xia, Yi Yang, Federico Longhini, Paolo Navalesi, Jennifer Beck, Christer Sinderby, Haibo Qiu

**Affiliations:** Department of Critical Care Medicine, Nanjing Zhongda Hospital, Southeast University, School of Medicine, 87 Dingjiaqiao Street, Nanjing, 210009 China; Department of Translational Medicine, Eastern Piedmont University “A. Avogadro”, Novara, Italy; Anaesthesia and Intensive Care, Sant’Andrea Hospital, ASL VC, Vercelli, Italy; CRRF Mons. L. Novarese, Moncrivello, VC Italy; Keenan Research Centre for Biomedical Science and Li Ka Shing Knowledge Institute of St. Michael’s Hospital, Toronto, Canada; Department of Pediatrics, University of Toronto, Toronto, Ontario M5G 1X8 Canada; Department of Medicine, University of Toronto, Toronto, Ontario Canada; Institute for Biomedical Engineering and Science Technology (iBEST) at Ryerson University and St-Michael’s Hospital, Toronto, Canada

## Abstract

**Introduction:**

Intrinsic positive end-expiratory pressure (PEEPi) is a “threshold” load that must be overcome to trigger conventional pneumatically-controlled pressure support (PS_P_) in chronic obstructive pulmonary disease (COPD). Application of extrinsic PEEP (PEEPe) reduces trigger delays and mechanical inspiratory efforts. Using the diaphragm electrical activity (EAdi), neurally controlled pressure support (PS_N_) could hypothetically eliminate asynchrony and reduce mechanical inspiratory effort, hence substituting the need for PEEPe. The primary objective of this study was to show that PS_N_ can reduce the need for PEEPe to improve patient-ventilator interaction and to reduce both the “pre-trigger” and “total inspiratory” neural and mechanical efforts in COPD patients with PEEPi. A secondary objective was to evaluate the impact of applying PS_N_ on breathing pattern.

**Methods:**

Twelve intubated and mechanically ventilated COPD patients with PEEPi ≥ 5 cm H_2_O underwent comparisons of PS_P_ and PS_N_ at different levels of PEEPe (at 0 %, 40 %, 80 %, and 120 % of static PEEPi, for 12 minutes at each level on average), at matching peak airway pressure. We measured flow, airway pressure, esophageal pressure, and EAdi, and analyzed neural and mechanical efforts for triggering and total inspiration. Patient-ventilator interaction was analyzed with the NeuroSync index.

**Results:**

Mean airway pressure and PEEPe were comparable for PS_P_ and PS_N_ at same target levels. During PS_P_, the NeuroSync index was 29 % at zero PEEPe and improved to 21 % at optimal PEEPe (P < 0.05). During PS_N_, the NeuroSync index was lower (<7 %, P < 0.05) regardless of PEEPe. Both pre-trigger (P < 0.05) and total inspiratory mechanical efforts (P < 0.05) were consistently higher during PS_P_ compared to PS_N_ at same PEEPe. The change in total mechanical efforts between PS_P_ at PEEPe_0%_ and PS_N_ at PEEPe_0%_ was not different from the change between PS_P_ at PEEPe_0%_ and PS_P_ at PEEPe_80%_.

**Conclusion:**

PS_N_ abolishes the need for PEEPe in COPD patients, improves patient-ventilator interaction, and reduces the inspiratory mechanical effort to breathe.

**Trial registration:**

Clinicaltrials.gov NCT02114567. Registered 04 November 2013.

## Introduction

Intrinsic positive end-expiratory pressure (PEEPi) refers to the increase in the end-expiratory elastic recoil pressure associated with an increase in lung volume above resting lung volume at end expiration consequent to dynamic hyperinflation. PEEPi impairs patient-ventilator interaction and efficiency of ventilatory assistance, increases inspiratory effort, causes dyspnea, and alters hemodynamics [1, 2].

In spontaneously breathing patients on conventional ventilatory assistance, PEEPi typically reveals itself as a delayed onset of assistance relative to the onset of neural inspiratory effort, where if ventilatory assistance is triggered on pressure, flow, or volume (i.e., pneumatic trigger), the PEEPi-induced threshold load must be overcome to initiate assistance [3]. Work in patients with chronic obstructive pulmonary disease (COPD) has demonstrated that application of external PEEP (PEEPe) can reduce the mechanical inspiratory effort [3–5].

In COPD patients receiving pressure support (PS) with pneumatic triggering and cycling-off, (PS_P_), the increased airway resistance prolongs the time constant and delays the cycling-off of ventilator support. Studies suggest a higher than conventional percentage of peak flow is required to adequately terminate assist; inappropriate settings for the cycling-off criteria are known to worsen dynamic hyperinflation and increase PEEPi [6, 7].

The effects of neural cycling-off of assistance in patients with COPD and PEEPi have not been evaluated during PS ventilation. Controlling ventilatory assistance by the diaphragm electrical activity (EAdi) - a neural signal - successfully improves patient-ventilator interaction during neurally adjusted ventilatory assistance (NAVA) compared to PS_P_ [8, 9]. A recent study has shown that the use of NAVA also leads to a decrease in the effort to trigger the ventilator, when compared to (PS_P_) [10]. These previous studies, however, compared a pressure-targeted mode (PS_P_) to a proportional mode (NAVA). Therefore, in the present study, we used neurally controlled PS (PS_N_), where the EAdi was used to initiate and terminate the breath, but with a targeted, fixed pressure. The primary objective was to show that PS_N_ can reduce the need for PEEPe to improve patient-ventilator interaction and to reduce both the pre-trigger and total inspiratory neural and mechanical efforts in COPD patients with PEEPi. A secondary objective was to evaluate the impact of applying PS_N_ on breathing pattern.

## Methods

The study was conducted in a 30-bed general intensive care unit (ICU) of a teaching hospital affiliated with Southeast University in China. The protocol was approved by Institutional Ethics Committee of Zhongda hospital (Approval Number: 2010ZDLL018.0), and informed consent was obtained from the patients or next of kin. The trial was registered at clinicaltrials.gov (NCT02114567).

### Patients

Twelve adult intubated and mechanically ventilated patients with early COPD and acute respiratory failure due to pneumonia were studied. COPD was defined as the patient having chronic cough, sputum or progressive dyspnea, and forced vital capacity rate of one second (FEV_1_/FVC) <0.7 after bronchodilation. Acute respiratory failure was defined as oxygenation index (PaO_2_/FiO_2_) <300 mmHg with or without elevated arterial carbon dioxide tension (PaCO_2_).

The inclusion criteria were: (1) static PEEPi ≥5 cm H_2_O (see below); (2) hemodynamic stability (heart rate <140 beats/minute, no vasopressors required, or <5 μg/kg/min dopamine); (3) no sedation or minimal analgesia with low dose of morphine (<3 mg/h, by continuous intravenous infusion); (4) breathing spontaneously but in need of partial ventilatory assistance, and (5) awake and able to positively cooperate, defined as the ability to follow an instruction (e.g., open their eyes, raise thumbs up, move limbs).

The exclusion criteria were: (1) tracheostomy; (2) treatment abandonment; (3) history of esophageal varices; (4) gastroesophageal surgery in the previous 12 months or gastroesophageal bleeding in the previous 30 days; (5) coagulation disorders (international normalized ratio >1.5 and activated partial thromboplastin time >44 s); (6) history of acute central or peripheral nervous system disorder or neuromuscular disease, and (7) lack of informed consent.

### Measurements

After obtaining consent, enrolled patients were switched to a Servo-i ventilator (Maquet, Solna, Stockholm, Sweden). A 16-F nasogastric feeding tube (NeuroVent Research Inc.; Toronto, ON, Canada) with electrodes measuring EAdi and balloons measuring esophageal (Pes) and gastric (Pga) pressures was inserted through the nose and secured after confirming positioning according to guidelines for NAVA catheter positioning (Maquet, Solna, Stockholm, Sweden). Flow and airway pressure (Paw) were acquired from the Servo-i ventilator whereas Pes and Pga were obtained via pressure transducers; all signals were digitized at 100 Hz and stored for offline analysis (NeuroVent Research Inc.; Toronto, ON, Canada). Mean arterial pressure (MAP) was measured with a blood pressure cuff (Philips G60).

### PS_P_ and PS_N_

#### Pneumatically controlled PS

Conventional pneumatically controlled PS (PS_P_) was used with the ventilator in the pressure support mode and was pneumatically triggered (flow-trigger 1 L/min) and cycled off (30 % of peak inspiratory flow). The rate of rise in pressure was set to 0.05 s in all patients.

#### Neurally controlled PS

Neurally controlled PS (PS_N_) was used with the ventilator in the NAVA mode, however, the NAVA level was set to maximum (NAVA level 15 cmH_2_O/μV) with upper pressure limits adjusted to achieve the targeted PS above PEEPe (same as PS_P_). PS_N_ was neurally triggered (EAdi trigger = 0.5 μV) and cycled off (70 % of peak EAdi).

### Study protocol

#### Determination of static PEEPi

Patients were initially on volume control ventilation (VCV) at zero PEEPe, tidal volume (V_T_) 6 mL/kg predicted body-weight (PBW), and inspiratory flow of 40 L/min, and mandatory breathing frequency (Bf) matching that observed during PS_P_ before sedation. To suppress the spontaneous drive to breathe (abolish EAdi), patients received continuous intravenous (IV) sedation by Propofol up to the dose of 2 mg/kg/h. If at this propofol dose the respiratory drive was not totally suppressed, Remifentanil was also infused at the dose of 6–15 μg/kg/h just before the measurement of compliance, resistance and static PEEPi. Static PEEPi was assessed during VCV at PEEPe of zero using the end-expiratory airway occlusion method [2]. PEEPe levels of 0 %, 40 %, 80 %, and 120 % of static PEEPi were then calculated and noted (subsequently referred to as PEEPe_0%_, PEEPe_40%_, PEEPe_80%_, and PEEPe_120%_). PEEPe was increased to determine the presence of expiratory flow limitation (EFL) [2].

#### Spontaneous breathing and return to PS at different levels of PEEPe

Sedation was discontinued and as spontaneous breathing and EAdi recovered, patients were returned to PS_P_ and adjusted to target 6 ml/kg (of PBW) and PEEPe of 5 cmH_2_O until a Ramsay score of 2–3 was obtained. This was followed by eight different ventilation periods: PS_P_ and PS_N_ at PEEPe_0%_, PEEPe_40%_, PEEPe_80%_, and PEEPe_120%_. First PS_P_ was applied targeting 6 ml/kg PBW with PEEPe levels randomized to be applied with either ascending or descending order. This was then repeated during PS_N_ with same PEEPe levels (as used with PS_P_) randomized to either ascending or descending order (independent of the order used during PS_P_). Assistance pressure above PEEPe was obtained by adjusting the upper pressure limit to the same assistance pressure (above PEEPe) that was observed for the corresponding PEEPe during the PS_P_ period. The average duration per PEEPe level was 12 (±1 SD) minutes. Arterial blood gases were measured at the end of each PEEPe level. Inspired fraction of oxygen (FiO_2_) was set similar to that at inclusion (Table 1) and not altered throughout the study.

**Table 1:** Patient demographics

Patient	Gender	Diagnosis	APACHE II	Days on MV	FiO_2_	C_RS_	R_RS_	PEEPi_STAT_	FEV1
					%	ml/cm H_2_O	cm H_2_O/l/s	cm H_2_O	% predicted
1	M	AECOPD, pneumonia, type 2 respiratory failure, pulmonary encephalopathy	32	5	40	33	19	6	56
2	M	AECOPD, pneumonia, type 2 respiratory failure	33	6	40	43	15	8	48
3	M	AECOPD, pneumonia, type 2 respiratory failure, septic shock	22	4	40	40	16	6	62
4	M	AECOPD, pneumonia, type 2 respiratory failure, pulmonary encephalopathy	34	4	40	42	18	5	41
5	M	AECOPD, pneumonia, type 2 respiratory failure, pulmonary encephalopathy	35	3	40	30	16	5	58
6	M	AECOPD, pneumonia, type 2 respiratory failure, pulmonary encephalopathy	33	2	40	26	16	6	55
7	M	AECOPD, pneumonia, type 2 respiratory failure, pulmonary encephalopathy	31	5	40	27	21	5	38
8	F	AECOPD, pneumonia, type 2 respiratory failure, pulmonary encephalopathy	32	5	40	31	27	5	47
9	M	AECOPD, pneumonia, type 2 respiratory failure, septic shock	42	8	40	42	23	5	46
10	M	AECOPD, pneumonia, type 2 respiratory failure, pulmonary encephalopathy	30	1	40	40	26	5	50
11	F	AECOPD, pneumonia, type 2 respiratory failure, pulmonary encephalopathy	31	2	40	23	24	6	37
12	F	AECOPD, pneumonia, type 2 respiratory failure, pulmonary encephalopathy	34	1	50	20	17	8	44
Mean			32.4	3.8	40.8	33.1	19.8	5.8	48.5
SD			4.5	2.1	2.9	8.1	4.2	1.1	8.0

*APACHE* acute physiology and chronic health evaluation, *AECOPD* acute exacerbation of chronic obstructive pulmonary disease, *MV* mechanical ventilation, *C*_*RS*_ compliance of respiratory system, *R*_*RS*_ resistance of respiratory system, *PEEPi*_*STAT*_ static intrinsic positive end expiratory pressure, *FEV1* forced expiratory volume in one second

### Data analysis

#### Parameters during volume control ventilation (and no spontaneous breathing)

Compliance was calculated from the formula:

Tidal volume/(Plateau pressure-total PEEP).

Resistance was calculated from the formula:

Resistance = (Peak pressure-Plateau pressure)/Flow.

EFL was determined from peak airway pressure during increase of PEEPe [2].

#### Respiratory parameters during spontaneous breathing on PS_P_ or PS_N_

The last 3 minutes of each condition were analyzed for the EAdi-derived, ventilator, and Pes-derived variables.

##### EAdi-derived variables

Neural inspiratory time (Ti_N_) was calculated between the onset of EAdi and the return to 70 % of peak EAdi. Neural expiratory time (Te_N_) was calculated as the time between the return to 70 % of peak EAdi and the onset of the next EAdi. We also calculated the neural duty cycle (Ti_N_/Tt_N_, where Tt_N_ = Ti_N_ + Te_N_), and neural breathing frequency (Bf_N_ = 60/Tt_N_). The peak inspiratory EAdi (ÊAdi) was calculated for the pre-trigger phase (ÊAdi_TRIG_), and for the entire inspiration (ÊAdi_TOT_).

##### Ventilator variables

PEEPe was measured as mean airway pressure in the expiratory state. V_T_ was obtained by flow integration. Mean airway pressure (P̅aw) was calculated during neural inspiration. Pneumatic inspiratory and expiratory times (Ti_P_ and Te_P_) were calculated from the airway pressure signal.

##### Pes-derived variables

The mean inspiratory change in Pes was calculated from onset of each inspiration (based on EAdi) for both the pre-trigger phase (ΔP̅es_TRIG_), as well as the total inspiration (pre-trigger effort included, ΔP̅es_TOT_). Transpulmonary pressure (P_L_) was calculated as Paw-Pes, and is presented for the total inspiration P_L_ (ΔP̅_LTOT_). Pre-trigger inspiratory pressure time product per minute was calculated for Pes (PTPes_TRIG_) as (ΔP̅es_TRIG_ × T_TRIG_ × Bf_N_), and for the total inspiration (PTPes_TOT_) as (ΔP̅es_TOT_ × Ti_N_ × Bf_N_). Neuromechanical efficiency (NME) was calculated for total inspiration as ΔP̅es/ÊAdi.

#### Analysis of patient-ventilator interaction

EAdi-to-trigger time difference in ms (T_TRIG_) was calculated between onset of EAdi and early initial rise in Paw. Cycling-off timing-difference in milliseconds (T_CYC-OFF_) was calculated between time points for early decrease in Paw and 30 % decline from EAdi peak.

Patient-ventilator interaction was evaluated by the NeuroSync Index, comparing Paw and EAdi waveforms with automated computer algorithms [11] and quantifying the error between them. Briefly, trigger and cycling-off errors were classified as either too early (negative values) or too late (positive values). Early and late triggering was defined as assistance starting before or after the onset of EAdi. Early and late cycling-off was defined as assistance starting before or after the return of EAdi to 70 % of its peak. Early trigger and cycling-off errors were normalized to the associated neural expiratory or inspiratory periods, respectively and presented in percent with a negative sign. Late trigger and cycling-off errors were normalized to related neural inspiratory or expiratory periods, respectively, and presented in percent with a positive sign. EAdi without associated assistance (ineffective effort) was defined as entire neural breathing cycles taking place without triggering assistance, and was assigned 100 % error. Assistance without associated EAdi (auto triggering) was defined as entire assistance cycles taking place without associated EAdi, and was assigned 100 % error. The NeuroSync index was calculated by averaging the errors for all events, the higher the NeuroSync index, the greater the error between EAdi and Paw.

### Statistics

Statistical analysis was performed with Sigma-Stat 3.5 (Jandel Scientific, California, USA). (Jandel Scientific, California, USA) Three types of comparisons were made: (i) within a mode, the impact of increasing PEEPe, (ii) at a given PEEPe, the impact of PS_P_ versus PS_N_, (iii) PS_N_ at PEEPe_0%_ versus PS_P_ at PEEPe_80%_ (considered to be optimal PEEP). Due to non-normally distributed data, we opted for within-subject comparison of all eight conditions using one-way repeated measures analysis of variance (ANOVA) on ranks and Student-Newman-Keuls test for post hoc analysis of multiple comparisons. Significant difference was defined as *P* <0.05. Power calculation suggested that a reduction of inspiratory effort by 50 % required 12 patients for a power of 1.0 with alpha of 0.05. To test if mechanical respiratory efforts were reduced similarly during PS_N_ without PEEPe and during PS_P_ with optimal PEEPe linear regression and Pearson product–moment correlation was used.

## Results

A total of 17 patients were screened; 5 did not meet the inclusion criteria of 5 cm H_2_O static PEEPi. Characteristics of the 12 enrolled patients are summarized in Table 1. All had acute exacerbation of COPD and type 2 respiratory failure. Six patients showed evidence of EFL. The mean age was 78.8 (SD ± 8.6) and body mass index (BMI) was 22.7 (SD ± 4.2). The last available forced expiratory volume in one second (FEV_1_) was 48.5 % (SD ± 8.0 %) predicted. The mean respiratory rate setting during VCV was 15 (SD ± 2) breaths per minute.

**Fig. 1:**
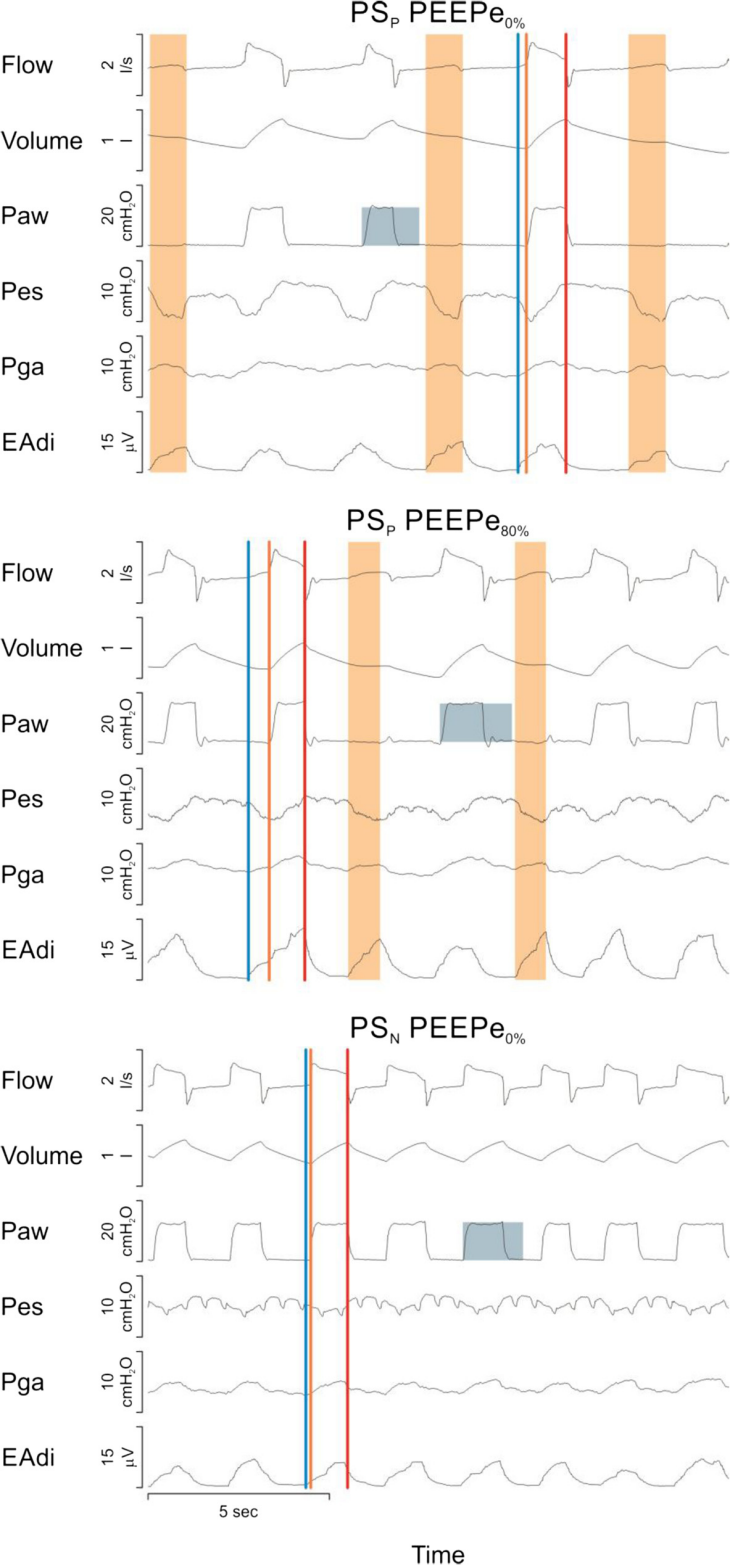
Time tracings from one individual patient. Time tracings of flow, volume, airway (*Paw*), esophageal (*Pes*) and gastric (*Pg*a) pressures, and diaphragm electrical activity (*EAdi*) during pneumatically triggered and cycled-off pressure support (*PS*_*P*_) at 0 % extrinsic positive end-expiratory pressure (*PEEPe*_*0%*_) (*top panel*), PS_P_ at *PEEPe*_*80%*_ (*middle panel*) and neurally triggered and cycled-off pressure support (*PS*_*N*_) at PEEPe_0%_ (*bottom panel*). *Blue*, *orange* and *red* lines indicate onset of diaphragm electrical activity (*EAdi*), nadir of Pes, and end of assistance, respectively. *Orange* bars indicate EAdi without assistance (ineffective efforts). A square wave pressure assistance profile of the same magnitude above positive end-expiratory pressure (*PEEP*) was obtained during all conditions (height of *blue box* same cm H_2_O in all panels). PS_N_ at PEEPe_0%_ synchronized the assistance and eliminated ineffective efforts. Pes was reduced by PS_P_ at PEEPe_80%_ and PS_N_ at PEEPe_0%_ whereas EAdi remained unchanged. The nadir of Pes occurred prior to the peak of EAdi and resulted in a positive inspiratory Pes deflection during all conditions, suggesting assistance delivery was too high in this subject. Note that Pes reverses from a negative to a positive trajectory as assistance starts, suggesting that assistance levels are excessive despite a tidal volume (*V*_*T*_) of 5.2 ml/kg of predicted body weight. Despite a low group-mean V_T_, this type of Pes waveform was noted in at least 50 % of the patients

Table 2 provides the ventilation parameters for the group, at different PEEPe levels, for PS_P_ and PS_N_. As per protocol design, P̅aw and PEEPe were matched for PS_P_ and PS_N_ at the same targeted levels (Table 2). The median time to peak pressure was 0.22 s (0.21–0.23, 25^th^–75^th^ percentile) in PS_P_, and was slightly shorter in PS_N_ (0.17 s, 0.15–0.22), the difference being 0.05 s (*P* = 0.04). Figure 1 shows an example of the time-tracings of flow, volume, Paw, Pes, Pga, and EAdi during PS_P_ at PEEPe_0%_ (top) and PEEPe_80%_ (middle) and during PS_N_ at PEEPe_0%_ (bottom) for one individual patient.

**Table 2:** Ventilation parameters, arterial blood gases, and mean arterial pressure at different PEEPe for PS_N_ and PS_P_

Parameter	Mode	PEEPe_0%_	PEEPe_40%_	PEEPe_80%_	PEEPe_120%_	P*
P̅aw (cm H_2_O)	PS_P_	10.9 (10.3, 11.3)	12.9 (12.3, 13.3)^a^	15.0 (14.8, 16.3)^ab^	17.4 (16.8, 18.3)^abc^	<0.001
	PS_N_	11.4 (10.8, 11.6)^x^	13.1 (12.1, 13.3)^a^	15.2 (14.4, 16.4)^ab^	17.5 (16.9, 18.6)^abc^	
PEEPe (cm H_2_O)	PS_P_	0.6 (0.4, 1.0)^z^	2.3 (2.2, 2.5)^az^	4.7 (4.2, 5.4)^ab^	7.1 (6.5, 7.5)^abc^	<0.001
	PS_N_	1.0 (0.5, 1.2)^x^	2.5 (2.3, 3.0)^a^	4.7 (4.4, 5.4)^ab^	7.0 (6.6, 7.5)^abc^	
V_T_ (ml/kg)	PS_P_	5.4 (4.2, 6.7)^z^	5.8 (4.9, 6.8)^z^	5.9 (5.0, 6.6)^abz^	5.5 (5.2, 7.0)^ab^	<0.001
	PS_N_	4.6 (4.3, 5.8)^x^	5.0 (4.4, 5.9)^a^	4.9 (4.6, 6.7)^ab^	5.0 (4.6, 7.1)^abc^	
Bf_N_ (breaths/min)	PS_P_	20.2 (17.7, 29.3)^z^	20.5 (17.6, 28.9)^z^	21.5 (17.2, 26.9)^z^	20.9 (17.9, 30.3)	0.003
	PS_N_	26.4 (17.3, 30.7)^x^	24.4 (17.2, 33.2)	22.1 (18.1, 31.5)^ab^	20.5 (16.0, 29.6)^ab^	
V_E_ (l/min)	PS_P_	6.05 (5.25, 8.13)^z^	6.54 (5.47, 8.91)^a^	7.05 (5.67, 9.02)^a^	7.07 (5.70, 11.62)^a^	<0.001
	PS_N_	6.43 (5.31, 9.54)^x^	6.92 (5.53, 9.98)^a^	7.14 (5.50, 9.20)^a^	7.79 (5.74, 9.66)^a^	
V_T_/Ti_P_ (ml/s)	PS_P_	424 (390, 446)^z^	429 (389, 472)	436 (386, 458)	438 (396, 466)	0.013
	PS_N_	414 (302, 491)^x^	443 (334, 508)^a^	452 (338, 526)^a^	479 (361, 513)^a^	
Ti_N_ (s)	PS_P_	0.90 (0.72, 1.03)	0.90 (0.74, 0.94)	0.84 (0.73, 0.98)	0.87 (0.69, 0.93)	NS
	PS_N_	0.84 (0.71, 0.96)	0.77 (0.71, 0.86)	0.81 (0.74, 0.86)	0.84 (0.73, 0.95)	
Te_N_ (s)	PS_P_	2.12 (1.43, 2.60)^z^	2.22 (1.45, 2.93)	2.17 (1.58, 3.14)^z^	2.13 (1.53, 2.58)	0.050
	PS_N_	1.76 (1.16, 2.66)^x^	2.09 (1.20, 2.77)^a^	2.02 (1.24, 2.59)^a^	2.14 (1.33, 2.92)^a^	
Ti_N_/Tt_N_ (%)	PS_P_	29.6 (27.5, 36.5)	28.7 (24.5, 39.0)	27.1 (23.4, 36.3)	26.1 (23.0, 36.7)	NS
	PS_N_	31.4 (25.6, 37.8)	29.4 (23.9, 38.5)	29.7 (24.6, 38.3)	30.6 (22.5, 36.8)	
pH	PS_P_	7.36 (7.33, 7.41)	7.38 (7.36, 7.41)^a^	7.39 (7.34, 7.44)^a^	7.39 (7.33, 7.42)^a^	0.009
	PS_N_	7.37 (7.33, 7.40)^x^	7.39 (7.34, 7.42)^a^	7.38 (7.32, 7.45)^a^	7.38 (7.35, 7.42)^a^	
PaCO_2_ (mm Hg)	PS_P_	37.9 (32.0, 48.6)	35.9 (31.2, 46.1)	36.3 (32.1, 46.8)	33.5 (32.9, 43.3)	NS
	PS_N_	40.4 (29.8, 47.5)	34.2 (31.2, 42.1)	35.4 (32.8, 41.8)	36.5 (30.7, 41.9)	
PaO_2_ (mm Hg)	PS_P_	84.0 (79.9, 113.9)	94.8 (84.9, 116.7)	98.7 (90.0, 124.3)	103.9 (84.0, 117.2)	NS
	PS_N_	89.7 (83.5, 122.9)	106.6 (81.2, 124.1)	99.4 (91.0, 123.9)	109.0 (95.0, 125.8)	
MAP (mm Hg)	PS_P_	89.0 (76.0, 92.7)	88.0 (77.0, 92.7)	86.7 (77.7, 91.7)	89.3 (79.0, 91.3)	NS
	PS_N_	88.3 (75.7, 91.3)	88.0 (75.3, 90.4)	88.7 (78.3, 91.0)	86.7 (80.3, 92.0)	

Values are presented as median (25–75 % interquartile range). *P values for one-way repeated measures analysis of variance on ranks for the eight conditions: within the same mode: ^a^ *P* <0.05 compared to PEEPe_0%_; ^b^ *P* <0.05 compared to PEEPe_40%_; ^c^ *P* <0.05 compared to PEEPe_80%._ Between modes: ^z^ *P* <0.05 compared to PS_N_ at same PEEPe. Comparison of PS_N_ zero PEEP to PS_P_ optimal PEEP: ^x^ *P* <0.05 PEEPe_0%_ at PS_N_ vs. PEEPe_80%_ at PS_P_. *PS*_*P*_ pneumatically triggered and cycled-off pressure support, *PS*_*N*_ neurally triggered and cycled-off pressure support ventilation, *P̅aw* mean airway pressure (including PEEPe), *PEEPe* extrinsic PEEP, *V*_*T*_ tidal volume, *V*_*E*_ minute ventilation, *Ti*_*N*_ neural inspiratory time, *Te*_*N*_ neural expiratory time, *Ti/Ttot*_*N*_ neural duty cycle, *Bf*_*N*_ neural breathing frequency, *PEEPi*_*STAT*_ static intrinsic positive end-expiratory pressure, *MAP* mean arterial pressure, *NS* not significant

### Patient-ventilator interaction

It can also be seen by the time tracings in Fig. 1, that patient-ventilator interaction was worse in PS_P_ than PS_N_. For the group (Table 3), both triggering (T_TRIG_) and cycling-off (T_CYC-OFF_) were consistently delayed during PS_P_, with delays at PEEPe_80%_ being 114 ms (trigger) and 118 ms (cycling-off) longer than during PS_N_ at PEEPe_0%_. Increasing from PEEPe_0%_ to PEEPe_80%_ during PS_P_ reduced the trigger delay by 93 ms (*P* <0.05) and delayed cycling-off by 54 ms. Timing of triggering and cycling-off was not affected by PEEPe during PS_N_.

**Table 3:** Patient ventilator interaction indices at different levels of PEEPe for PS_P_ and PS_N_

Parameter	Mode	PEEPe_0%_	PEEPe_40%_	PEEPe_80%_	PEEPe_120%_	P*
T_TRIG_ (ms)	PS_P_	276 (169, 370)^z^	198 (156, 357)^az^	183 (143, 312)^abz^	154 (33, 236)^abcz^	<0.001
	PS_N_	69 (56, 82)^x^	54 (32, 70)	59 (17, 82)	76 (54, 90)	
T_CYC-OFF_ (ms)	PS_P_	76 (21, 36)^z^	126 (44, 401)^az^	130 (47, 432)^abz^	106 (14, 314)^abcz^	<0.001
	PS_N_	12 (8, 14)^x^	8 (2, 14)	5 (−2, 12)	2 (−7, 10)	
NeuroSync index (%)	PS_P_	29.1 (13.9, 46.8)^z^	25.3 (15.5, 40.9)^az^	20.6 (13.5, 37.1)^abz^	17.7 (10.5, 38.9)^abcz^	<0.001
	PS_N_	5.6 (4.2, 7.8)^x^	6.0 (4.9, 8.3)	6.2 (5.1, 8.5)	6.7 (3.8, 10.3)	
EAdi without assist (%)	PS_P_	0.8 (0, 10.1)	1.1 (0, 18.7)	0 (0, 10.8)	0 (0, 11.4)	<0.001
	PS_N_	0 (0, 0)	0 (0, 0)	0 (0, 0)	0 (0, 0)	
Synchrony (inside box) (%)	PS_P_	4.7 (0, 16.3)^z^	7.8 (0, 20)^az^	11.5 (0, 37.4)^abz^	7 (0.2, 46.3)^abcz^	<0.001
	PS_N_	89.4 (76.2, 97.9)^x^	91.3 (76, 95.7)	88 (78, 93)	80 (63.4, 97.4)	
Dyssynchrony (outside box) (%)	PS_P_	83.5 (76.3, 92)^z^	79.3 (63, 86.7)^az^	76.9 (62, 87.5)^abz^	70.9 (52.3, 89.3)^abcz^	<0.001
	PS_N_	9 (2.1, 23.1)^x^	7.3 (1.8, 21.1)	10.8 (6.5, 17.4)	18.6 (2.1, 32.7)	

Values are presented as median (25–75 % interquartile range). *P values for one-way repeated measures analysis of variance on ranks for the eight conditions: within the same mode: ^a^ *P* <0.05 compared to extrinsic positive end-expiratory pressure (PEEPe)_0%_; ^b^ *P* <0.05 compared to PEEPe_40%_; ^c^ *P* <0.05 compared to PEEPe_80%_. Between modes: ^z^ *P* <0.05 compared to PS_N_ at same PEEPe. Comparison of PS_N_ zero PEEP to PS_P_ optimal PEEP: ^x^ *P* <0.05 PEEPe_0%_ at PS_N_ vs. PEEPe_80%_ at PS_P_. *PS*_P_ pneumatically triggered and cycled-off pressure support, *PS*_*N*_ neurally triggered and cycled-off pressure support ventilation, *PEEPe* extrinsic PEEP, *T*_*TRIG*_ diaphragm electrical activity (EAdi)-to-trigger time difference, *T*_*CYC-OFF*_ cycling-off timing difference

**Fig. 2:**
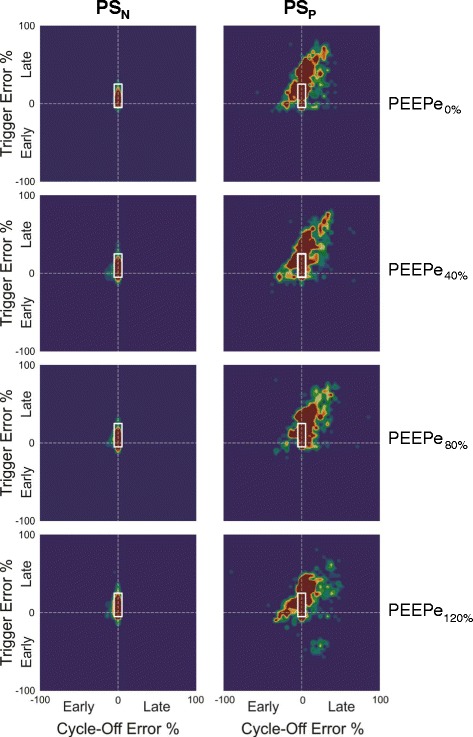
Patient-ventilator interaction for all patients represented topographically. Topographic distribution of triggering error (y-axis) and cycling-off error (x-axis). The red area indicates 80 % of the most frequent patient-ventilator interactions for all breaths in all subjects during neurally triggered and cycled-off pressure support (*PS*_*N*_) (*left panels*) and pneumatically triggered and cycled-off pressure support (*PS*_*P*_) (*right panels*) during extrinsic positive end-expiratory pressure (*PEEPe*)_0%_, PEEPe_40%_, PEEPe_80%_, and PEEPe_120%_, (*top to bottom*). See text for details

Figure 2 shows the topographic distribution of timing errors for triggering (y-axis) and cycling-off (x-axis), respectively, for all patients. The red area indicates 80 % of the most frequent patient-ventilator interactions for all breaths in all subjects during PS_N_ (left panels) and PS_P_ (right panels) during PEEPe_0%_, PEEPe_40%_, PEEPe_80%_, and PEEPe_120%_, (top to bottom). During PS_N_ at PEEPe_0%_ triggering was concentrated within an area ranging from minus 5 % to 25 % for triggering error (Y-axis) and minus 5 % to 5 % error during cycling-off (x-axis) regardless of PEEPe (indicated by box).

During PS_P_, there was a widespread variability ranging from about minus 10 % to 60 % for triggering error (y-axis) and about minus 30 % to 30 % error for cycling-off (x-axis), regardless of PEEPe. Regardless of PEEPe, less than 10 % of breaths landed within the box during PS_P_, compared to more than 80 % during PS_N_ (Table 3).

During PS_N_ at PEEPe_0%,_ 52 % (SD ± 30 %) of all breaths were terminated by neural cycling-off at 70 % of peak EAdi and at this point the inspiratory flow had decreased to 46 % (SD ± 19 %) of peak flow. The remaining 48 % (SD ± 30 %) of breaths were terminated earlier due to pressure exceeding the upper pressure limit by 3 cm H2O causing the −5 % cycling-off errors indicated in Fig. 2, left panel.

During PS_N_, the NeuroSync index was consistently lower, indicating improved patient ventilator interaction, at all levels of PEEPe. Increasing PEEPe improved patient-ventilator interaction i.e., decreased NeuroSync index during PS_P_ but had no effect during PS_N_ (Table 3). Regarding severe asynchronies, EAdi without trigger (ineffective efforts) exceeded 10 % in three patients (12 %, 12 % and 20 %) during PS_P_ (Table 3). Other asynchronies were not frequent during either PS_P_ or PS_N_.

### Neural (EAdi variables) and mechanical (Pes variables) effort

**Fig. 3:**
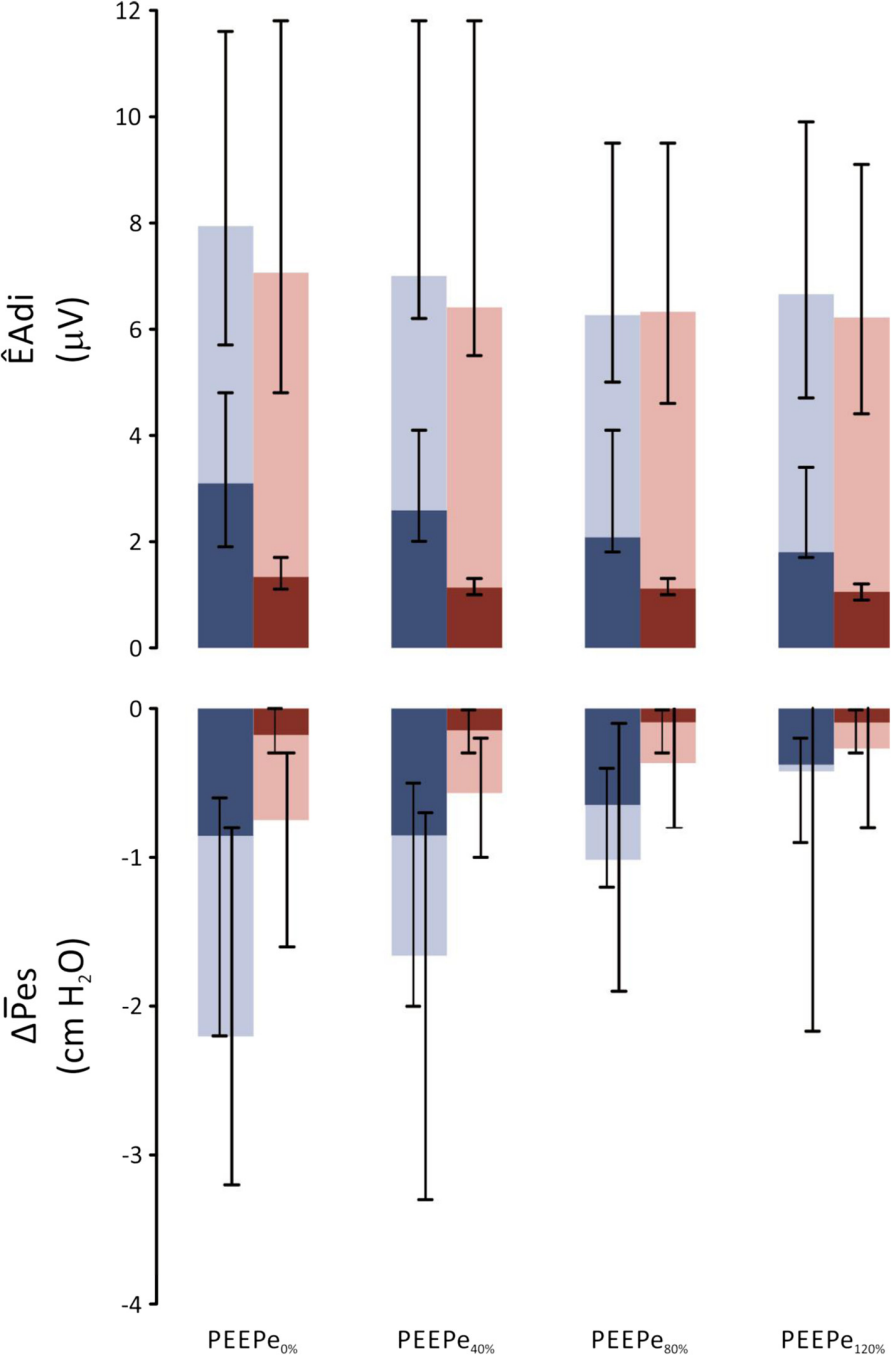
Neural and mechanical effort during neurally triggered and cycled-off pressure support (*PS*_*N*_) and pneumatically triggered and cycled-off pressure support (*PS*_*P*_) at different levels of extrinsic positive end-expiratory pressure (*PEEPe*). *Top panel*: peak diaphragm electrical activity (*ÊAdi*, *y-axis*) for PS_P_ (*blue bars*) and PS_N_ (*red bars*) for triggering (*darker bars*) and total inspiration (*darker bars + lighter bars*) with increasing PEEPe (*x-axis*). *Bottom panel*: mean inspiratory deflection in esophageal pressure (*ΔP̅es*, *y-axis*) for PS_P_ (blue bars) and PS_N_ (*red bars*) for triggering (*darker bars*) and total inspiration (*darker bars + lighter bars*) with increasing PEEPe (*x-axis*). Median and interquartile ranges are presented. For clarity, a corresponding statistical description is provided in Table 4

Figure 3 shows the neural and mechanical effort for triggering and for the whole inspiration in all subjects, at all PEEPe levels, for PS_N_ and PS_P_. The corresponding statistics are provided in Table 4 for clarity. ÊAdi_TRIG_ was lower during PS_N_ than PS_P_ at all PEEPe levels. During PS_N_, ÊAdi_TRIG_ at PEEPe_0%_ was also lower compared to PS_P_ at PEEPe_80%_ (Table 4). Increasing PEEPe decreased ÊAdi_TRIG_ during both PS_P_ and PS_N_. ÊAdi_TOT_ was not significantly between PS_P_ and PS_N_, nor did it change with changing PEEPe.

**Table 4:** Neural and mechanical indices of respiratory effort at different levels of PEEPe during PS_P_ and PS_N_

Parameter	Mode	PEEPe_0%_	PEEPe_40%_	PEEPe_80%_	PEEPe_120%_	P*
Δ P̅es_TRIG_ (cm H_2_O)	PS_P_	−0.9 (−2.2, −0.6)^z^	−0.9 (−2.0, −0.5)^z^	−0.6 (−1.2, −0.4)^abz^	−0.4 (−0.9, −0.2)^abcz^	<0.001
	PS_N_	−0.2 (−0.3, −0.0)^x^	−0.1 (−0.3, −0.0)	−0.1 (−0.3, −0.0)	−0.1 (−0.3, −0.0)	
Δ P̅es_TOT_ (cm H_2_O)	PS_P_	−2.2 (−3.2, −0.8)^z^	−1.7 (−3.3, −0.7)^z^	−1.0 (−1.9, −0.1)^abz^	−0.4 (−2.2, 0.2)^acz^	<0.001
	PS_N_	−0.7 (−1.6, −0.3)	−0.6 (−1.0, −0.2)^a^	−0.4 (−0.8, 0.4)^ab^	−0.3 (−0.8, 0.4)^abc^	
PTPes_TRIG_ (cm H_2_O*s/min)	PS_P_	−5.0 (−23.8, −2.7)^z^	−3.3 (−19.4, −1.5)^z^	−2.4 (−10.5,−1.1)^abz^	−1.3 (−4.2, −0.3)^abcz^	<0.001
	PS_N_	−0.2 (−0.6, −0.1)^x^	−0.2 (−0.4, −0.0)	−0.0 (−0.3, 0.1)	−0.1 (−0.4, 0.0)	
PTPes_TOT_ (cm H_2_O*s/min)	PS_P_	−44.5 (−81.3, −13.9)^z^	−29.7 (−58.3, −11.5)^z^	−17 (−34.2, −2.6)^abz^	−7.3 (−49.8, 3.0)^abcz^	<0.001
	PS_N_	−15.8 (−27.7,−5.9)	−11.5 (−16.7, −4.9)^a^	−7.5 (−15.4, 5.5)^ab^	−5.1 (−14.1, 4.2)^abc^	
ÊAdi_TRIG_ (μV)	PS_P_	3.1 (1.9, 4.8)^z^	2.6 (2.0, 4.1)^z^	2.1 (1.8, 4.1)^abz^	1.8 (1.7, 3.4)^abcz^	<0.001
	PS_N_	1.3 (1.1, 1.7)^x^	1.1 (1.0, 1.3)^a^	1.1 (1.0, 1.3)^ab^	1.0 (0.9, 1.2)^ab^	
ÊAdi_TOT_ (μV)	PS_P_	7.9 (5.7, 11.6)	7.0 (6.2, 11.8)	6.3 (5.0, 9.5)	6.7 (4.7, 9.9)	<0.001
	PS_N_	7.1 (4.8, 11.8)	6.4 (5.5, 11.8)	6.3 (4.6, 9.5)	6.2 (4.4, 9.1)	
Δ P̅es/Δ P̅_L_ (%)	PS_P_	24.5 (35.4, 11.0)^z^	18.2 (29.8, 8.3)^z^	12.5 (22.7, 1.3)^abz^	6.6 (21.1, −2.70)^abcz^	<0.001
	PS_N_	7.0(12.1, 2.9)^x^	5.4 (6.9, 2.2)	2.8 (7.6, −4.5)^ab^	2.2 (7.6, −5.1)^abc^	
NME (cm H_2_O/μV)	PS_P_	−0.27 (−0.34, −0.16)^z^	−0.22 (−0.32, −0.11)^az^	−0.13 (−0.28, −0.03)^abz^	−0.09 (−0.15, 0.02)^abcz^	<0.001
	PS_N_	−0.11 (−0.22, −0.04)	−0.07 (−0.15, −0.03)^a^	−0.06 (−0.10, 0.04)^ab^	−0.05 (0.08, 0.06)^abc^	

Values are presented as median (25–75 % interquartile range). *P values for one-way repeated measures analysis of variance on ranks for the eight conditions: •within the same mode: ^a^ *P* <0.05 compared to PEEPe_0%_; ^b^ *P* <0.05 compared to PEEPe_40%_; ^c^ *P* < 0.05 compared to PEEPe_80%_. Between modes: ^z^ *P* <0.05 compared to PS_N_ at same PEEPe comparison of PS_N_ zero PEEP to PS_P_ optimal PEEP: ^x^ *P* <0.05 PEEPe_0%_ at PS_N_ vs. PEEPe_80%_ at PS_P._ *PS*_*P*_ pneumatically triggered and cycled-off pressure support, *PS*_*N*_ neurally triggered and cycled-off pressure support ventilation, *ΔP̅es*_*TRIG*_ pre-trigger mean deflection of esophageal pressure, *ΔP̅es*_*TOT*_ total inspiratory mean deflection for esophageal pressure, *PTPes*_*TOT*_ total inspiratory pressure time product per minute for esophageal pressure, *PTPes*_*TRIG*_ pre-trigger pressure time product per minute for esophageal pressure, *ÊAdi*_*TOT*_ peak inspiratory diaphragm electrical activity (*EAdi*) for total inspiration, *ÊAdi*_*TRIG*_ peak pre-trigger EAdi, *ΔP̅es/ΔP̅*_*L*_ esophageal pressure contribution to transpulmonary pressure during inspiration, *NME* neuromechanical efficiency calculated for esophageal pressure

**Fig. 4:**
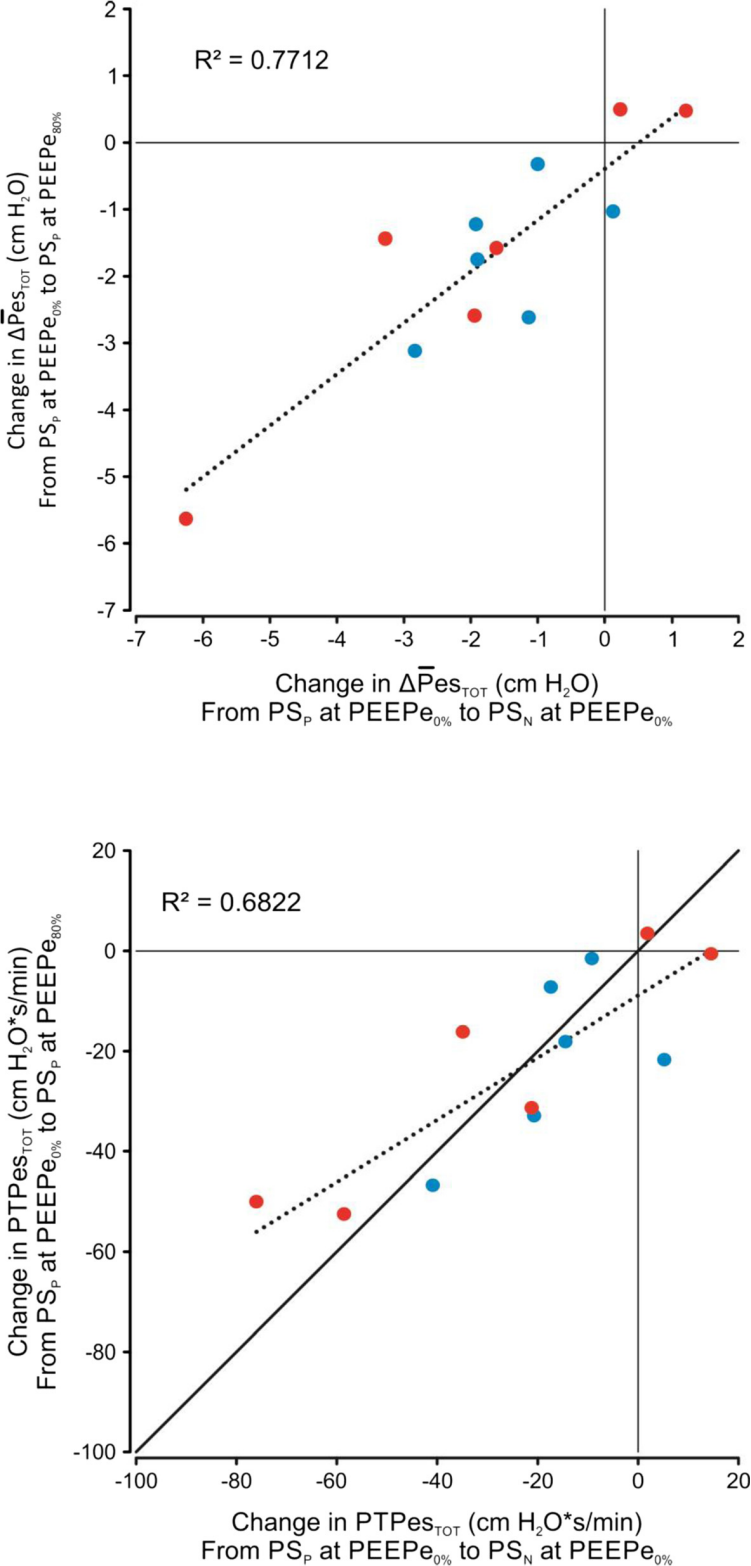
Comparison of the changes in mechanical effort for all subjects. Changes in total mechanical effort from pneumatically triggered and cycled-off pressure support (*PS*_*P*_) at extrinsic positive end-expiratory pressure (*PEEPe*)_0%_ to neurally triggered and cycled-off pressure support (*PS*_*N*_) at PEEPe_0%_ (*x-axes*) and PS_P_ at PEEPe_80%_ (*y-axes*). *Upper panel*: inspiratory esophageal pressure deflection (ΔP̅es_TOT_) per breath; *lower panel*: inspiratory pressure time product per minute (*PTPes*_*TOT*_). *Blue symbols* indicate patients with expiratory flow limitation; *red symbols* indicate patients without expiratory flow limitation. See text for details

Both ΔP̅es_TRIG_ and ΔP̅es_TOT_ were higher during PS_P_ compared to PS_N_ at the same PEEPe (Fig. 3 and Table 4): ΔP̅es_TRIG_ was consistently and markedly reduced during PS_N_ compared to PS_P_. Increasing PEEPe reduced ΔP̅es_TRIG_ during PS_P_ at PEEPe_80%_ but did not change during PS_N_: ΔP̅es_TOT_ decreased with increasing PEEPe during both PS_P_ and PS_N_. Four patients had positive ΔP̅es_TOT_ during PS_P_ at PEEPe_80%_ and one patient had positive ΔP̅es_TOT_ during PS_N_ at PEEPe_0%_. Figure 4 shows that the change in total mechanical efforts between PS_P_ at PEEPe_0%_ and PS_N_ at PEEPe_0%_ (x-axis) is similar to the change between PS_P_ at PEEPe_0%_ and PS_P_ at PEEPe_80%_ (with strong correlation: *R*^2^ = 0.77 for ΔP̅es_TOT_ and *R*^2^ = 0.68 for PTPes_TOT_).

ΔP̅es/ΔP̅_L_ ranged between 2.2 and 24.5 %, decreased with increasing PEEPe during both PS_P_ and PS_N_, and was lower during PS_N_ (Table 4).

### Neuromechanical efficiency (NME)

Neuromechanical efficiency at the same PEEPe was lower during PS_N_ compared to PS_P_ and decreased with increasing PEEPe during both PS_P_ and PS_N_ (Table 4). There was no difference in NME between PS_N_ at PEEPe_0%_ and PS_P_ at PEEPe_80%_. The reduction in NME from PEEPe_0%_ to PEEPe_80%_ during PS_P_ was not different (*P* = 0.699) for patients with EFL (median 28 %, 25^th^–75^th^ percentile 14–46 %) and without EFL (41, 14–75 %).

## Discussion

This study shows that neurally controlled pressure support improves patient-ventilator interaction, nearly abolishes pre-trigger inspiratory neural and mechanical effort, and shows - even when zero PEEP is applied - similar total inspiratory neural and mechanical effort as conventional pressure support with an optimal PEEPe. The main strength of the study is that it is the first to show that both neural monitoring and neural control of patient-ventilator interaction in patients with PEEPi are superior to pneumatic monitoring and pneumatic control of pressure support.

### Patient-ventilator interaction

In agreement with previous studies [5, 10, 12–16], increasing PEEPe during PS_P_ reduced the trigger delay. As hypothesized, the EAdi trigger-synchronization nearly abolished both neural and mechanical pre-trigger efforts regardless of PEEPe. Our results showing that both neural and mechanical pre-trigger efforts were reduced with increasing PEEPe during PS_P_ confirms that application of PEEPe counteracts PEEPi and reduces pre-trigger mechanical effort with pneumatic triggering [5], although not as efficiently as during neural triggering, similar to the recent work of Bellani [10].

Cycling-off assistance in PS mode is conventionally based on the relative reduction in inspiratory flow. This algorithm is an oversimplification and not physiologically sound, as flow during ventilatory assistance is influenced by multiple factors, such as respiratory system time constant, neural inspiratory time, level of pressure support, and inspiratory muscle pressure [17]. In the present study, the cycling-off setting was 30 % of peak inspiratory flow (default setting of the ventilator utilized). This choice could be critiqued as being too low in sensitivity in COPD patients as percentages from 40 to 70 % have been suggested as more feasible [6, 7]. As there are no guidelines on how to adjust cycling-off for each individual patient, we opted to stay within default settings.

In support of cycling-off at 70 % of peak EAdi being feasible was our finding that assistance was either EAdi-terminated when flow corresponded to 46 % of peak flow or immediately before EAdi termination due to the inspiratory muscle relaxation increasing pressure in the circuit by 3 cm H_2_O above the targeted pressure (Servo-I manual). Thus the PS_N_ cycling-off in the present study coincides with suggested flow cycling-off at 40–70 % of peak flow in COPD [6, 7].

PS_N_ showed high precision of triggering and cycling-off of pressure relative to the neural effort and centered 80 % of breaths within a narrow range of error (Fig. 2). In contrast, PS_P_ showed poor precision of both triggering and cycling-off relative to the neural effort. Although group median values indicated delays (Table 3), the topographical distribution of all breaths and in all subjects shows that ventilatory assistance could start and cycle off prematurely. It is questionable if adjustment of trigger and cycling-off settings during PS_P_ could have corrected this extreme heterogeneity of timing of assist relative to neural inspiratory effort.

With regards to the overall patient-ventilator interaction, a low NeuroSync index and little inter-individual variability, confirms the effectiveness of PS_N_ to synchronize assistance in the presence of PEEPi. Although improved by increasing PEEPe, the NeuroSync index was at least three times higher (worse patient-ventilator interaction) during PS_P_ mainly due to dys-synchrony i.e., trigger and cycling-off errors, which cannot be determined with pressure-flow-volume waveform analysis without EAdi [11, 18]. The low incidence of other asynchronies e.g., ineffective efforts and auto-triggering is in agreement with previous work by Thille [19] showing that limiting V_T_ (6 ml/kg) - as in the present study - improves patient ventilator interaction during PS_P_. However, three patients (25 %) approached a high frequency of EAdi-without-assistance (ineffective efforts) during PS_P_ which is recognized as severe asynchrony and associated with adverse outcomes such as increased duration of mechanical ventilation [20, 21].

With regards to the effort throughout the entire inspiration, our results (Fig. 4) indicating that reductions from PEEPe_0%_ during PS_P_ to PEEP_0%_ during PS_N_ equaled those from PEEPe_0%_ during PS_P_ to PEEP_80%_ during PS_P_, support our assumption that synchronized assist overcomes PEEPi in COPD patients without the need to apply PEEPe. Thus, neural triggering allows a unique starting point for assistance delivery during every breath regardless of hyperinflation and PEEPi, however, the subsequent inspiration requires that the combined patient effort and assistance (i.e., the transpulmonary pressure) is sufficient to overcome the respiratory system’s resistive and elastic forces. In contrast, PS_P_ and fixed PEEPe only compensate for the estimated average increase in elastic recoil at end-expiration due to dynamic hyperinflation and cannot correct for breath-by-breath changes in PEEPi.

Our results that applying PEEPe during PS_P_ reduced mechanical effort for the entire inspiration agree with previous studies [4, 5]. A curious observation of the present study was that the reduction in total inspiratory mechanical effort from PEEP_0%_ to PEEP_80%_ during PS_P_ was larger than what could be explained by the reductions in pre-trigger mechanical effort. Even more confounding, increasing PEEPe actually reduced total inspiratory mechanical effort during PS_N_; a decrease that could not be attributed to reductions in the pre-trigger mechanical effort, as it was already abolished by the neural triggering. A likely possibility for why the mechanical efforts decreased is that PEEPe induced hyperinflation (increased end expiratory lung volume) which would explain the reduction in NME (less pressure for a given neural output).

This is the first study measuring the effect of PEEPe on neural effort strictly in COPD patients (the work of Bellani [10] included 50 % COPD patients). Although our results showed that pre-trigger neural effort could be reduced by increasing PEEPe during PS_P_, the total neural inspiratory effort did not reach a significant decrease with increasing PEEPe during PS_P_ nor during PS_N_. This supports our thought that reduced total inspiratory mechanical effort with increasing PEEPe were in part associated with hyperinflation-induced respiratory muscle weakness [22], and not de-activation of the muscles. Previous studies indicate that application of CPAP (Continuous Positive Airway Pressure) in COPD patients with PEEPi increases end-expiratory lung volume [4, 23]. However, it has been suggested that application of PEEPe below the level of PEEPi in patients with EFL does not increase hyperinflation [24]. Our results did not indicate a difference for NME between patients with and without EFL. However, the present study showed a reduction in NME with increasing PEEPe during both PS_P_ and PS_N_, which could have been attributed to impaired contractility due to hyperinflation [22]. Thus, our finding that the total inspiratory mechanical effort during PS_N_ at PEEPe_0%_, matched PS_P_ at PEEPe_80%_ suggests that neural triggering is at least as efficient as titration of PEEPe to overcome PEEPi, and reduce total inspiratory mechanical effort. However, both methods pay a toll in terms of reduced NME.

It is important to point out that several patients received too high assistance (approximately 10 cm H_2_O PS above PEEPe), resulting in low values of total inspiratory mechanical effort (<2.5 cm H_2_O Pes), suggesting that the patient’s contribution to tidal volume was very low. At this high level of unloading, further reduction in neural inspiratory effort is limited [25–27], which could explain the modest decrease in total neural inspiratory effort.

Despite relatively low V_T_ targeted in the present study, which should contradict the notion of over-assistance [19], PaCO_2_ values were low, suggesting that certain patients could have been subjected to hyperventilation. Yet, another factor to explain the low total inspiratory mechanical effort could be respiratory muscle weakness. A limitation was that we did not evaluate respiratory effort sensation or dyspnea, which could have added insight to the issue of PEEPe and assistance levels that were too high. Note that with PS_N_, the amount of pressure support delivered should be greater than PEEPi. If the initial pressure delivery is not adequate to counteract PEEPi, the elastic recoil in the system would cause an increase in airway pressure [10] (see Fig. 4 in that report), and would activate the cycling-off (pressure algorithm) used with neural control of PS (as the safety algorithm).

Despite PEEPe being demonstrated to reduce PEEPi and work of breathing, many factors of how to implement PEEPe are unclear [4]. It is not clear whether PEEPi should be expressed in terms of its dynamic PEEPi or static PEEPi components. In spontaneously breathing, mechanically ventilated patients with active expiration there are currently no methods available to reliably determine the optimal level of static PEEPi and there is ongoing evaluation of reliability in different methods determining dynamic PEEPi [12, 28–30]. Moreover, the implementation of bias flow for the use of flow-trigger creates further complication as it underestimates dynamic PEEPi [31]. Maltais et al. [32] reported that in paralyzed patients, dynamic PEEPi underestimates static PEEPi due to regional differences of mechanical properties within the lungs. We therefore opted to measure static PEEPi during VCV in the absence of spontaneous breathing effort.

One limitation of the present study was that we could not randomize PS_P_ and PS_N_, because PS_P_ had to be adjusted first (with a target tidal volume of 6 ml/kg), in order to be matched with the upper pressure limits that were obtained during PS_N_. We did, however, randomize the ascending or descending order of the applied PEEPe in both arms, albeit we acknowledge that randomizing all PEEPe levels would be preferred. Due to risk of the steps between PEEPe levels being too large we decided not to randomize the order in which PEEPe was applied, but to apply PEEPe in either progressively increasing or decreasing order.

## Conclusion

The present study shows that PS_N_ overcomes the need for PEEPe to overcome PEEPi in COPD patients. PS_N_ improves patient-ventilator interaction and reduces inspiratory mechanical effort to breathe. Although the present study suggests that PS_N_ (at zero PEEP) can efficiently replace PS_P_ with optimal PEEPe, use of PEEPe for other reasons, e.g., alveolar recruitment, would of course still apply. The clinical importance of improving patient-ventilator interaction in COPD remains to be studied.

## Key messages

- Neurally controlled pressure support ventilation is feasible in patients with COPD demonstrating intrinsic PEEP
- Neurally controlled pressure support, compared to conventional, pneumatically controlled pressure support, improves patient-ventilator interaction and reduces inspiratory effort, even in the absence of external PEEP
- Neurally controlled pressure support overcomes the need for extrinsic PEEP, in order to overcome intrinsic PEEP in COPD patients

## Abbreviations

Bf: breathing frequency
Bf_N_: neural breathing frequency
COPD: chronic obstructive pulmonary disease
CV: coefficient of variation
EAdi: diaphragm electrical activity
ÊAdi: peak inspiratory EAdi
ÊAdi_TRIG_: peak pre-trigger EAdi
EFL: expiratory flow limitation
FEV_1_: forced expiratory volume in one second
FEV_1_/FVC: forced vital capacity rate of one second
MAP: mean arterial pressure
NAVA: neurally adjusted ventilatory assist
NME: neuromechanical efficiency
PaCO_2_: arterial carbon dioxide tension
PaO_2_/FiO_2_: oxygenation index
Paw: airway pressure
P̅aw: mean airway pressure
PEEPe: extrinsic positive end-expiratory pressure
PEEPe_0%_: PEEPe levels of 0 % of PEEPi_STAT_
PEEPe_120%_: PEEPe levels of 120 % of PEEPi_STAT_
PEEPe_40%_: PEEPe levels of 40 % of PEEPi_STAT_
PEEPe_80%_: PEEPe levels of 80 % of PEEPi_STAT_
PEEPi: intrinsic positive end-expiratory pressure
PEEPi_STAT_: Static intrinsic positive end-expiratory pressure
Pes: esophageal pressure
Pga: gastric pressure
P_L_: transpulmonary pressure
PS_N_: neurally triggered and cycled-off pressure support
PS_P_: pneumatically triggered and cycled-off pressure support
PTPes_TOT_: total inspiratory pressure time product per minute for esophageal pressure
PTPes_TRIG_: pre-trigger pressure time product per minute for esophageal pressure
T_CYC-OFF_: cycling-off timing-difference
Te_N_: Neural expiratory time
Te_P_: ventilator (pneumatic) expiratory time
Ti/Tt_N_: neural duty cycle
Ti_N_: neural inspiratory time
Ti_P_: ventilator (pneumatic) inspiratory time
Tt_N_: neural respiratory cycle time
T_TRIG_: EAdi-to-trigger time difference
VVC: volume control ventilation
V_T_: tidal volume
ΔP̅es_TOT_: total inspiratory mean deflection for esophageal pressure
ΔP̅es_TRIG_: pre-trigger mean deflection of esophageal pressure
ΔP̅_LTOT_: total inspiratory mean deflection for transpulmonary pressure
